# RNA Sequencing Analysis of Molecular Basis of Sodium Butyrate-Induced Growth Inhibition on Colorectal Cancer Cell Lines

**DOI:** 10.1155/2019/1427871

**Published:** 2019-02-27

**Authors:** Qianwen Zhou, Guiqin Li, Siyu Zuo, Wenjing Zhu, Xiaoqin Yuan

**Affiliations:** ^1^Department of Anatomy, Histology and Embryology, Nanjing Medical University, Nanjing 211166, China; ^2^Department of Gastroenterology, People's Hospital of Lianshui, Huai'an 223400, China; ^3^Key Laboratory for Aging & Disease, Nanjing Medical University, Nanjing 211166, China

## Abstract

Butyrate is a short-chain fatty acid decomposed from dietary fiber and has been shown to have effects on inhibition of proliferation but induction of apoptosis in colorectal cancer cells. However, clinical trials have yielded ambiguous outcomes with regard to its antitumor activities. In this study, we aimed to explore the molecular mechanisms underlying the sensitivity of colorectal cancer cells to sodium butyrate (NaB). RNA sequencing was used to establish the whole-transcriptome profile in NaB-treated versus untreated colorectal cancer cells. Differentially expressed genes were bioinformatically analyzed to predict their possible involvement in NaB-triggered cell death, and the expression of eight dysregulated genes was validated by quantitative real-time PCR. We found that there were a total of 7192 genes (5720 upregulated and 1472 downregulated, fold-change ≥ 2 or ≤ 0.5 for upregulation or downregulation, q-value < 0.05) differentially expressed in NaB-treated cells as compared with the untreated controls. Gene Ontology and Kyoto Encyclopedia of Genes and Genomes pathway analysis demonstrated that the differentially expressed genes were enriched in DNA replication, cell cycle, homologous recombination, pyrimidine metabolism, mismatch repair, and other signaling pathways and may take part in NaB-induced cell death. Among the identified factors, the MCM2-7 complex might be a target of NaB. Our findings provide an important basis for further studies of the complicate network that might regulate sensitivity of colorectal cancer cells to NaB.

## 1. Introduction

Colorectal cancer (CRC) is the third most common cancer and the fourth leading cause of cancer-related death worldwide [1]. Although targeted therapeutics such as cetuximab and bevacizumab have been developed and other treatment modalities advanced, the prognosis for patients with CRC of high metastatic potential remains poor [2]. Therefore, new chemoprophylactic agents are still urgently needed for prevention of CRC at their early stages.

Previous studies have established that a high fiber diet is effective in the prevention of some gastrointestinal cancers [3, 4]. The fermentation of dietary fibers by bacteria in the human colon gives rise to short-chain fatty acids (SCFAs) mainly including butyrate (4C), propionate (3C), and acetate (2C) at a molar ratio of 60:25:15 [5], all of which are indispensable to keep colonic mucosa healthy and maintain an energy source for colonocytes. More importantly, SCFAs were shown capable of inducing tumor cell death and are currently being evaluated as adjunctive chemotherapeutic agents for CRC [6]. In the human intestine, it is estimated that the physiological concentrations of total SCFAs range from 70 to 140 mM in the proximal colon but from 20 to 70 mM in the distal colon and from 20 to 40 mM in the distal ileum [7]. Intriguingly, the mode of SCFA-induced cell death is dependent on the carbon length. For instance, butyrate has a preference for induction of apoptosis or necrosis in gastric cancer cells compared with propionate [6].

Given that butyrate exerts potent and pleiotropic effects on multiple cancers, it has attracted the most attention as a tumor-suppressive molecule. Butyrate caused massive apoptosis in HT-29 CRC cells and rendered primary adenocarcinoma CRC cells less invasive [6]. Sodium butyrate (NaB) was also shown to halt the progression of cell cycle, promote cell differentiation, or elicit apoptosis and autophagy in various cancer cells such as CRC, lymphoma, and breast cancer cells [8–12]. While normal colonocytes also make use of butyrate as one of their preferred energy sources [13], butyrate appears to accumulate more in cancerous colonocytes owing to the Warburg effect, in which it could function as a histone deacetylase (HDAC) inhibitor that results in inhibition of cell proliferation and stimulation of apoptosis [14]. In CRC cells, NaB appears to induce endoplasmic reticulum stress and autophagy by markedly enhancing the expression levels of endoplasmic reticulum (ER) stress-associated proteins such as BIP, CHOP, PDI, and IRE-1*α* [12]. Accumulating evidences from a number of experimental and human studies have also suggested that the mechanism by which butyrate suppresses malignant cell proliferation and reduces cancer risk may act through its ability to neutralize the function of some oncogenic miRNAs [15–17]. However, the complex molecular mechanisms and signaling pathways underlying the regulation of apoptosis and growth inhibition in cancer cells by butyrate remain to be elucidated.

In this study, we explored the global changes in gene expression in CRC cells following NaB treatment using next-generation RNA sequencing (RNA-Seq) to examine variations at the transcriptome level and identify differentially expressed genes (DEGs). These findings will provide important data resources for further investigation of the molecular mechanisms and signaling pathways associated with NaB antitumor activities in colorectal cancer cells.

## 2. Materials and Methods

### 2.1. Cell Lines and Reagents

HCT116 and SW480 CRC cell lines were maintained in RPMI1640 (HyClone, South Logan, Utah, USA) and DMEM (HyClone), respectively, containing 10% FBS (Gibco, Grand Island NY, USA), 100 U/ml penicillin, and 0.1 mg/ml streptomycin (Gibco). NaB (Sigma-Aldrich, St. Louis, MO, USA) was dissolved in PBS (2 M stock solution) and used at the indicated concentration.

### 2.2. Cell Proliferation Assay

3 × 10^3^ HCT116 and SW480 cells in 100 *μ*l complete media with different concentrations of NaB or transfected with specific siRNAs were seeded into 96-well plates and cultured in an incubator containing 5% CO_2_ at 37°C. At 0, 24, 48, 72, and 96 h after drug exposure, cell viability was determined by Cell Counting Kit 8 (Donjindo, Kumamoto, Japan). The absorbance was measured at the wavelength of 450 nm using a microplate reader (Bio-Tek, Winooski, VT, USA). The specific siRNAs

### 2.3. Cell Cycle Assay

SW480 and HCT116 cells were exposed to 2 mM NaB for 24 h and harvested by washing in ice-cold PBS and resuspending in 80% ethanol for 2 h. Cells were then treated with RNase (100 *μ*g/ml) and propidium iodide (50 *μ*g/ml). After a 30-min incubation at 37°C, cells were subjected to fluorescence-activated cell sorting (FACS) using a FACSCalibur flow cytometer (BectonDickinson, Heidelberg, Germany).

### 2.4. Cell Apoptosis Assay

SW480 and HCT116 cells were treated with 2 mM NaB for 24 h or HCT116 cells were transfected with specific siRNAs and the rate of apoptosis was measured by using the Annexin V-FITC/PI Kit (Keygen, Nanjing, China) in accordance with the manufacturer's protocol.

### 2.5. RNA Extraction and Purification

Total RNA was isolated by TRIzol reagent (Life Technologies, Carlsbad, CA, USA) according to the manufacturer's instructions and Agilent Bioanalyzer 2100 (Agilent Technologies, Santa Clara, CA, USA) was used to evaluate RNA integrity. Qualified RNA was further purified with the RNAClean XP Kit (Beckman Coulter, Fullerton, CA, USA) and RNase-Free DNase Set (QIAGEN, Hilden, Germany).

### 2.6. Library Construction for RNA-Seq and the Sequencing Procedures

Paired-end libraries were constructed by the TruSeq® RNA Sample Preparation Kit (Illumina, San Diego, CA, USA) following the TruSeq® RNA Sample Preparation Guide. Briefly, poly-T oligo-attached magnetic beads were used to purify the poly-A containing mRNA molecules. After the purification, fragmentation of the mRNA into small pieces was accomplished by exposure to divalent cations for 8 min at 94°C. The fragmented RNAs were reverse-transcribed into the first strand cDNA by reverse transcriptase and random primers, and the second strand cDNA were synthesized using DNA Polymerase I and RNase H, followed by an end repair process with the addition of a single “A” base and ligation of the adapters. The final cDNA library was created after purification and enrichment with PCR and the purified libraries were quantified and validated by the Qubit® 2.0 Fluorometer (Life Technologies) and the Agilent 2100 Bioanalyzer (Agilent Technologies), respectively, to confirm the insert and calculate the concentration. Clusters were generated by cBot from the library which was diluted to 10 pM and sequenced on the Illumina HiSeq 2500 (Illumina).

### 2.7. Data Analysis for Gene Expression

Levels of gene expression were presented as FPKM (fragments per kilobase of exon per million fragments mapped) [18]. The false discovery rate (FDR) was applied to set the p-value significance threshold in multiple tests [19] and the fold changes estimated based on the FPKM in each sample. The filter criteria of FDR ≤0.05 and fold-change ≥2 were used to determine the differentially expressed genes.

### 2.8. Functional Annotation Analysis

The biological relevance of unique genes in the significant or representative profiles of DEGs (The Gene Ontology Consortium, 2000) was analyzed by Gene Ontology (GO) whose annotations were downloaded from the Gene Ontology (http://www.geneontology.org/), UniProt (http://www.uniprot.org/), and NCBI (http://www.ncbi.nlm.nih.gov/). The significant GO categories were identified by Fisher's exact test with* P* < 0.05, which means that significantly overrepresented genes in the set of DEGs assigned to a specific functional category are more than expected by chance. Significant pathways of the DEGs were analyzed and identified according to the Kyoto Encyclopedia of Genes and Genomes (KEGG) database.

### 2.9. Quantitative RT-PCR (qPCR)

RNA was extracted from HCT116 or SW480 cells treated with 2 mM NaB for 24 h with TRIzol reagent (Life Technologies) by the manufacturer's protocol. Reverse transcription was accomplished with the PrimeScript™ 1st Strand cDNA Synthesis Kit (Takara Bio, Shiga, Japan). Real-time PCR was performed using SYBR green reagents (Takara Bio) in an ABI 7300 sequence detector (Applied Biosystems, Foster City, CA, USA) to determine the relative expression of genes of interest. The sequences of the PCR primers used are present in Supplementary Table S1. The 18S gene served as the internal control.

### 2.10. Small Interfering RNA Transfection

Small interfering RNAs (siRNAs) targeting MCM2, MCM3, MCM5, MCM7, and negative control siRNA were synthesized by RiboBio (Guangzhou, China). The siRNA sequences were as follows: MCM2 siRNA: sense: 5′-AGGUAAUUUCUAGAUAGCAAUGU-3′, antisense: 5′ACAUUGCUAUCUAGAAAUUACCU-3′; MCM3 siRNA: sense: 5′-GCAUUGUCACUAAAUGUUCUCUAGU-3′, antisense: 5′-ACUAGAGAACAUUUAGUGACAAUGC-3′; MCM5 siRNA: sense: 5′-GGAUGAACUCAAGCGGCAU-3′+dTdT, antisense: 5′-AUGCCGCUUGAGUUCAUCC+dTdT-3′; MCM7 siRNA: sense: 5′-GAGUUGGUGGACUCAAUUU+dTdT-3′, antisense: 5′-AAAUUGAGUCCACCAACUC+dTdT-3′

### 2.11. Western Blotting Analysis

Lysates from HCT116 and SW480 cells treated with or without 2 mM NaB were run on SDS-PAGE, transferred to polyvinylidene fluoride (PVDF) membranes, and blotted with primary antibodies directed against GAPDH (Abcam, Cambridge, UK), MCM2, MCM3, MCM5, or MCM7 (all from Santa Cruz Bio, Dallas, TX, USA), followed by respective secondary antibodies and chemiluminescent detection.

### 2.12. Statistical Analysis

All data are expressed as mean ± SEM. Student's t-test and one-way ANOVA were employed for comparisons.* P* < 0.05 was considered statistically significant.

## 3. Results

### 3.1. Sodium Butyrate-Induced Cell Cycle Arrest and Apoptosis of Colon Cancer Cells

**Figure 1:**
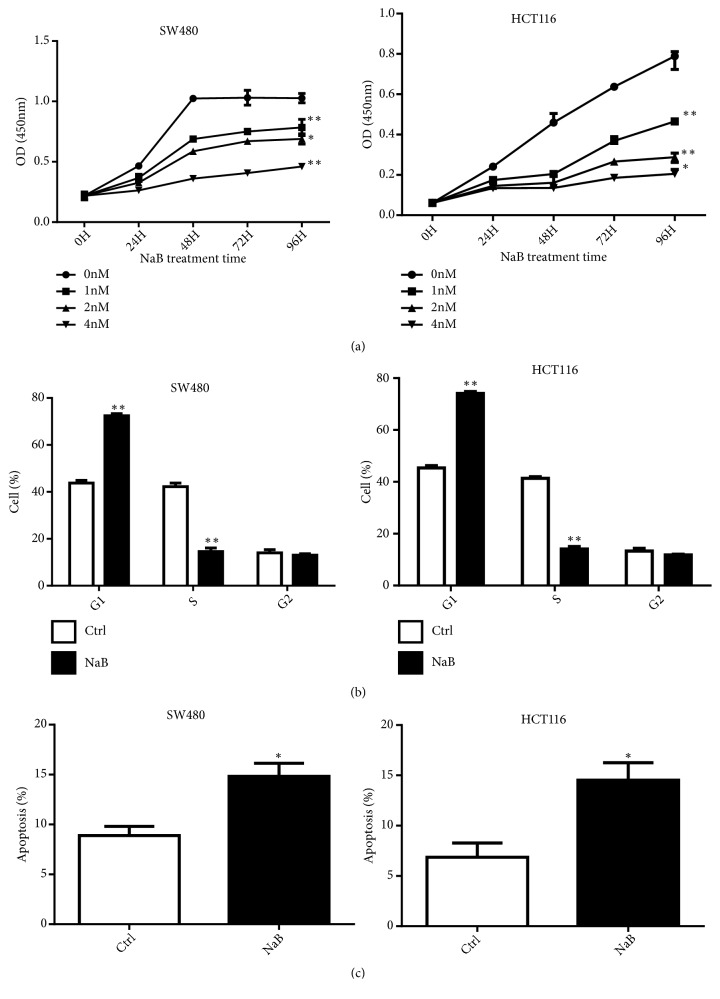
NaB inhibited proliferation of CRC cells. (a) The cell proliferation of SW480 (left panel) and HCT116 cells (right panel) treated with different doses and exposure times of NaB was examined by CCK8 assay. (b) The cell cycle of SW480 (left panel) and HCT116 cells (right panel) treated with 2 mM NaB for 24 h. (c) Apoptotic rates in SW480 (left panel) and HCT116 cells (right panel) treated with 2 mM NaB for 24 h. Data are presented as mean ± SD; *∗P* < 0.05; *∗∗P* < 0.001.

To verify the antiproliferative effect of NaB on colon cancer cells, we performed CCK-8 assays to measure the growth curve of SW480 and HCT116 cells treated with increasing concentrations of NaB. As shown in Figure 1(a), NaB inhibited the proliferation of SW480 and HCT116 cells in a dose-dependent fashion. Since exposure to 2 mM NaB for 24 h produced approximately 40% inhibition of cell proliferation, we selected this concentration for subsequent experiments. Analysis of cell cycle phase distribution and apoptotic rates showed that 2 mM NaB treatment for 24 h resulted in G1 arrest and increased apoptosis in SW480 and HCT116 cells (Figures 1(b) and 1(c)).

### 3.2. Sequencing Results on RNA-Seq Analysis

To explore the underlying mechanisms by which NaB exerted its antiproliferative effect on CRC cells, we performed RNA-Seq analysis to comprehensively define the transcriptomic changes in CRC cells after NaB exposure. We established six cDNA libraries including Ctrl1, Ctrl2, and Ctrl3 from SW480 cells treated with PBS as control and Test1, Test2, and Test3 from SW480 cells treated with 2 mM NaB for 24 h. Among the six samples, the raw sequencing reads were in the ranges of 62,935,944 to 76,897,270. For each library the sequencing depth reached as saturated as 40 M reads (Supplementary Figure S1). After the low-quality reads were filtered out, 94.5% of the original reads remained (Supplementary Table S2). Alignment of the reads to the reference genome indicated that approximately 97% of the reads mapped uniquely to the human genome (Table 1).

### 3.3. Identification of DEGs

**Figure 2:**
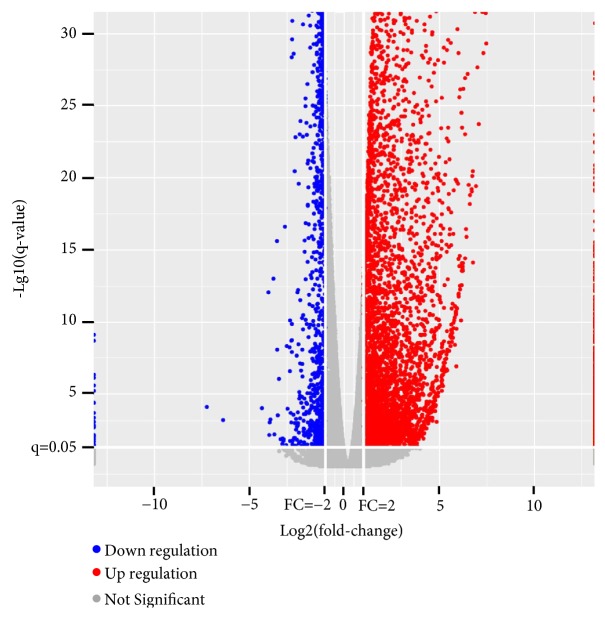
Volcano plot of differentially expressed genes in NaB-treated SW480 cells. The log⁡2 fold-change difference is represented on the x-axis and -log⁡10 of corrective p-value (q-value) is represented on the y-axis. Red and blue plots show upregulated and downregulated genes, respectively.

The DEGs were obtained by comparing all test samples (marked with NaB) against all the control samples (marked with Ctrl). We found that 7192 genes were significantly altered in expression, including 5720 upregulated and 1472 downregulated genes (with a fold-change ≥2 or ≤0.5, q-value < 0.05). For an overview of the differentially expressed genes, volcano plots were drawn where the fold-change of gene expression was plotted on the* x*-axis versus the significance of difference in gene expression between pools on the* y*-axis (Figure 2). Furthermore, to identify genes better associated with the effect of NaB, we filtered the above 7192 DEGs with a stringency cutoff:* p* < 0.001, FPKM≥1 for upregulated genes in the test group, and FPKM≥1 for downregulated genes in the control group. This resulted in 3067 DEGs, including 1968 upregulated genes and 1099 downregulated genes, and these were subjected to further analysis (Supplementary Table S3).

### 3.4. Analyses of GO Functional Enrichment from DEGs and KEGG Pathway

**Figure 3:**
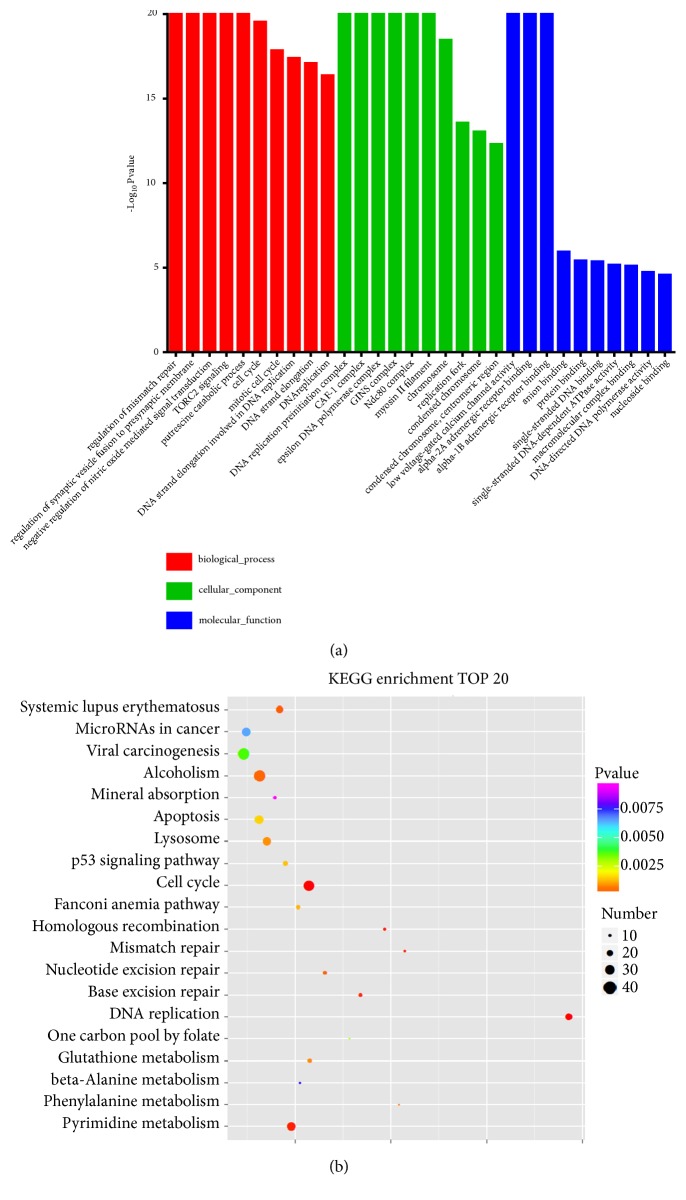
Gene ontology (GO) and KEGG pathway enrichment. (a) The top 10 GO terms with most significant* P* values for biological processes (red), cellular component (green), and molecular function (blue). The top 20 significant pathways are shown in (b).

We clustered the 3067 DEGs based on the GO and KEGG analysis. According to the significance, we obtained the top 10 clusters in biological processes, cellular components, and molecular functions (Figure 3(a)). The GO functional enrichment analysis demonstrated that DEGs were significantly enriched in the functional categories associated with the regulation of mismatch repair, cell cycle, DNA replication preinitiation complex, GINS complex, and replication fork, among other categories. Pathway analysis was also performed to further identify critical signal regulation pathways using the KEGG database, and the top 20 significant KEGG pathways were identified as shown in Figure 3(b). These pathways were significantly related to DNA replication, cell cycle, homologous recombination and mismatch repair, among others (*P* ≤ 0.01).

### 3.5. NaB Treatment Downregulated Gene and Protein Levels of the MCM2-MCM7 Complex

**Figure 4:**
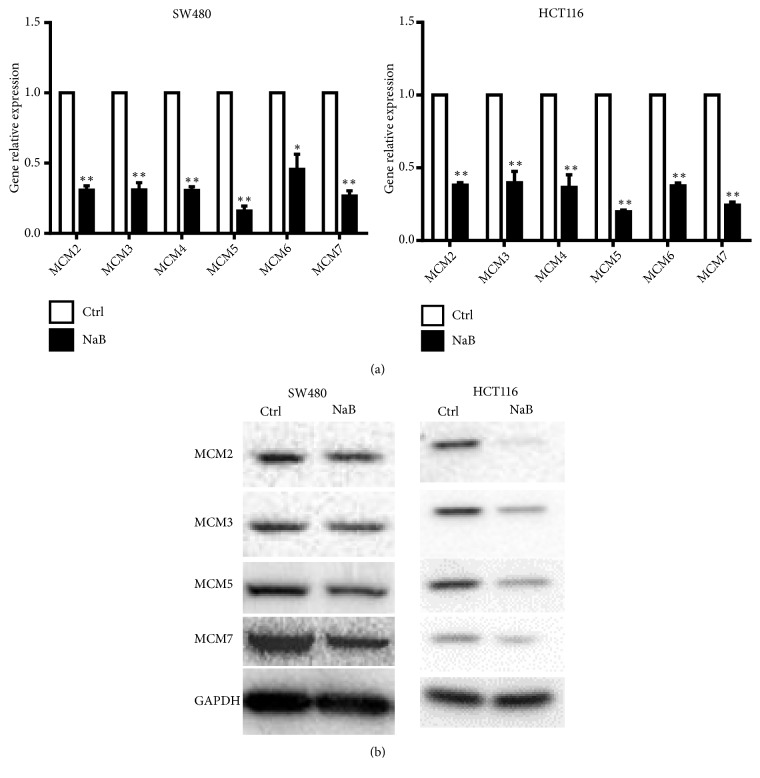
MCM2-MCM7 complex expression was validated by qPCR and Western blotting. (a) MCM complex genes in untreated cells and cells treated with 2 nM NaM for 24 h were validated with real-time PCR. (b) MCM complex protein expressions in SW480 (left) and HCT116 cells (right) treated with 2 nM NaM for 24 h were validated with Western blotting. Data are presented as mean ± SD. *∗P* < 0.05; *∗∗P* < 0.001.

**Figure 5:**
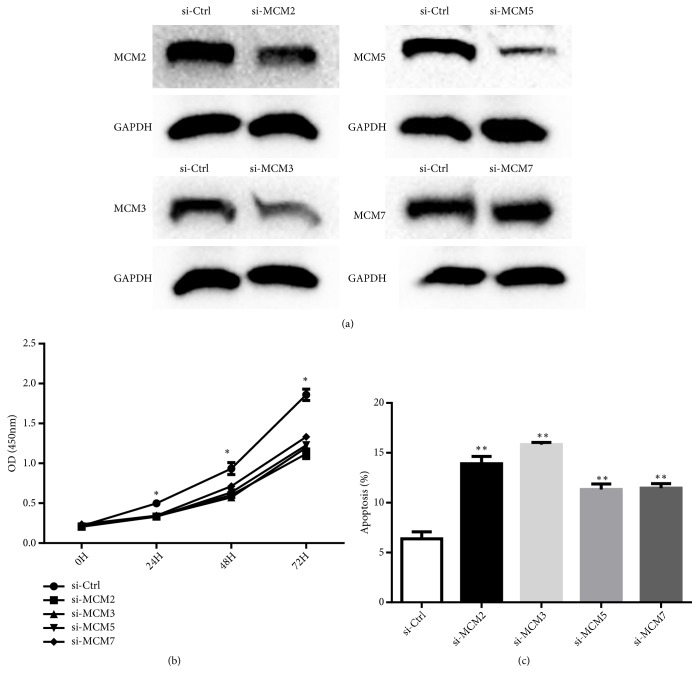
MCM2-7 knockdown inhibited proliferation of HCT116 cells. (a) MCM2, MCM3, MCM5, and MCM7 siRNA were transfected into HCT116 cell and their expressions were detected by Western blot. HCT116 cells with knockdown of MCM2, MCM3, MCM5, and MCM7 were examined by CCK8 assay (b) and by apoptotic assay (c). Data are presented as mean ± SD; *∗P* < 0.05; *∗∗P* < 0.001.

Our RNA-Seq data showed that the MCM2-MCM7 complex was downregulated in response to NaB in CRC cells. The MCM2-MCM7 complex, which includes MCM2, MCM3, MCM5, and MCM7, is thought to be the first replisome component that arrives at replication initiation sites and is critical for replisome assembly during DNA replication. To verify the RNA-Seq data, we examined the expressions of MCM2-MCM7 mRNAs in control and NaB-treated cells using qPCR. As shown in Figure 4(a), MCM2-MCM7 mRNAs were significantly downregulated in NaB-treated cells. The relative fold-changes from qRT-PCR analysis were similar to the RNA-Seq results, indicating the dependability of the RNA-Seq platform. We also tested the expression of MCM2-MCM7 proteins by Western blot analysis showing that MCM2, MCM3, MCM5, and MCM7 proteins were decreased in NaB-treated cells (Figure 4(b)), which confirmed the qPCR results. In order to conform the effect of MCM2-MCM7 on cell proliferation and apoptosis, HCT116 cells were transfected with siRNA specific to MCM2, MCM3, MCM5, and MCM7 (Figure 5(a)) and measured with CCK8 and cell apoptosis assay, respectively. The results showed that knockdown of MCM2, MCM3, MCM5, and MCM7 inhibited HCT116 cell proliferation and promoted HCT116 cell apoptosis (Figures 5(b) and 5(c)). These results indicated that NaB may exhibit antitumor activities in CRC cells via downregulating the MCM2-MCM7 complex to impair DNA replication assembly.

## 4. Discussion

Butyrate is a SCFA that is generated by the gut microbiota in the process of anaerobic fermentation of dietary fibers. A previous study showed that butyrate can inhibit tumor progression through inhibition of HDAC thus inducing apoptosis in cancer cells [20]. However, the precise molecular pathway by which butyrate induces apoptosis and inhibits growth in cancer cells remains elusive. In the present study, we demonstrated that NaB causes cell cycle arrest and apoptosis in colon cancer cells via regulation of genes associated with DNA replication and repair.

Butyrate has been evaluated in clinical trials as a HDAC inhibitor for treating human cancer [21, 22]. It has been shown that butyrate inhibited tumor cell proliferation and induced apoptosis by the route of the intrinsic apoptosis pathway through modulation of reactive oxygen species in a variety of cancer types including CRC [23–26]. While butyrate is capable of inhibiting cell proliferation and inducing apoptosis, there is very little information on the mechanisms regulating this process. Here we demonstrated that NaB can significantly inhibit the proliferation and trigger apoptosis in SW480 and HCT116 cells. We then examined this effect on a more global level by performing RNA-Seq on cells after 24 h treatment with 2 mM NaB. We identified 7192 DEGs between the NaB-treated group and the control group, with 5720 upregulated and 1472 downregulated genes. To identify genes better associated with the function of NaB, a stringency cutoff (p < 0.001, FPKM ≥1 for upregulated genes in the test group, and FPKM ≥1 for downregulated genes in the control group) was used to identify 3067 DEGs, including 1968 upregulated genes and 1099 downregulated genes. GO annotation and enrichment analysis showed that DEGs participate in a wide variety of functions that include mismatch repair, cell cycle, DNA replication preinitiation complex, GINS complex, and replication fork. Functional annotation demonstrated that the DEGs largely affect the DNA repair capacity occurring in the progress of transcription regulation. How the targets of each GO term are involved in the sensitivity of CRC cells to NaB should be examined in further studies.

To explore which pathway may involve in NaB-induced cell death, KEGG pathway analysis was employed to authenticate pathways and illustrate the biological importance of significant DEGs. The results indicated that the DEGs were enriched for several pathways, including DNA replication, cell cycle, homologous recombination, pyrimidine metabolism, and mismatch repair, among others. A majority of those pathways have not been previously studied in the context of NaB-induced cancer cell death, with only very few exceptions. For example, one study showed that butyrate suppresses tumor growth and induces apoptosis by regulation of expression of HDAC, SIRT-1, caspase 3, and NF*κ*B [20]. In addition to butyrate, several other HDAC inhibitors have also been found to be helpful in complementing chemotherapy strategies aimed to halt cancer cell proliferation and growth eventually leading to cell death [27, 28].

Besides butyrate, other SCFA such as acetate is also reported to exert antiproliferative effects on cancer. Sodium acetate inhibited CRC cell proliferation and induced apoptosis by increasing permeabilization of lysosomal membrane and subsequent release of cathepsin D [29]. It has also been demonstrated that acetate could induce the glioma stem cell (GSC) growth arrest via affecting glycolytic metabolism [30, 31]. In addition to SCFA, medium-chain fatty acid (MCFA; C8:0-14:0) also possessed the anticancer property as evidenced by an induction of cell cycle arrest and apoptosis of Caco-2 cells through reducing GSH availability and generation of ROS [32].

The MCM complex consists of MCM2–7 subunits and is known to be highly conserved from yeast to humans. This complex acts as the licensing system that gathers both the replication machinery at initiation origins and the catalytic core of the CMG (Cdc45-Mcm2-7-GINS) helicase that facilitate unwinding DNA for elongation [33]. Studies have shown that MCM2-MCM7 is one of major cellular regulatory targets relevant to cancer development and progression [33, 34]. Expression of MCM2 and MCM7 are positively correlated with the Ki67 proliferation marker in prostate cancer, breast cancer, colon cancer, ovarian cancer, lung, and bladder cancer [34–36]. Moreover, some tumor suppressors and regulatory proteins such as the Rb tumor suppressor family and CDK inhibitors can bind to MCM7 and block MCM2-7 activity [37, 38]. Several MCM2-7-targeting small molecule inhibitors such as widdrol have been recently discovered [39]. In present study, we found knockdown of MCM2, MCM3, MCM5, and MCM7 inhibited cell proliferation and induced cell apoptosis. Our data showed that the antitumor effects of NaB in CRC cells may involve its targeting the MCM2-7 complex.

## 5. Conclusions

Our study identified differentially expressed mRNAs in CRC cells treated with NaB. Our results and analyses provide a global view of the function of the differentially expressed mRNAs. Most of the molecular and pathway abnormalities discovered in this study have not been previously reported to be associated with NaB sensitivity in CRC cells. The MCM2-7 complex might be one of the targets of NaB in CRC cells. Since the dysregulated genes may participate in NaB growth-inhibitory effects via diverse mechanisms, further investigation is required to explore the role of these transcripts, pathways, and the interaction networks in determining NaB efficacy.

## Figures and Tables

**Table 1:** Sample mapping results.

Samples_ID	All reads	Mapped reads	Mapped Pair Reads	Mapped broken-pair reads	Mapped Unique reads	Mapped Multi reads	Mapping ratio
Crtl1	68,875,890	67,126,346	66,000,392	1,125,954	66,866,741	259,605	97.46%
Crtl2	71,377,408	69,590,420	68,415,488	1,174,932	69,321,293	269,127	97.50%
Crtl3	56,507,850	55,041,342	54,066,584	974,758	54,803,408	237,934	97.40%
Test1	70,595,792	68,730,746	67,566,036	1,164,710	68,472,382	258,364	97.36%
Test2	59,002,178	57,493,455	56,522,938	970,517	57,282,175	211,280	97.44%
Test3	66,821,272	65,071,593	63,935,532	1,136,061	64,821,951	249,642	97.38%

## Data Availability

Our all data used to support the findings of this study are included within the article and the supplementary information files.

## Supplementary Materials

Supplementary 1
* Figure S1*: saturation analysis of the RNA sequencing data from six libraries. Ctrl1, Ctrl2, and Ctrl3 from SW480 and Test1, Test2, and Test3 from SW480 treated with 2 nM NaB 24 hours. x-axis, sequencing depth; y-axis, proportion of covered genes.

Supplementary 2
* Table S1*: specific primers used for qPCR.

Supplementary 3
* Table S2*: quality metrics for the RNA-Seq data.

Supplementary 4
* Table S3*: differentially expressed genes detected by RNA-Seq.
