# Supplementary figures and images for: RNA Sequencing Analysis of Molecular Basis of Sodium Butyrate-Induced Growth Inhibition on Colorectal Cancer Cell Lines
Source: Biomed Res Int. 2019 Feb 27;2019:1427871. doi: 10.1155/2019/1427871 (PMC6415300; doi:10.1155/2019/1427871)

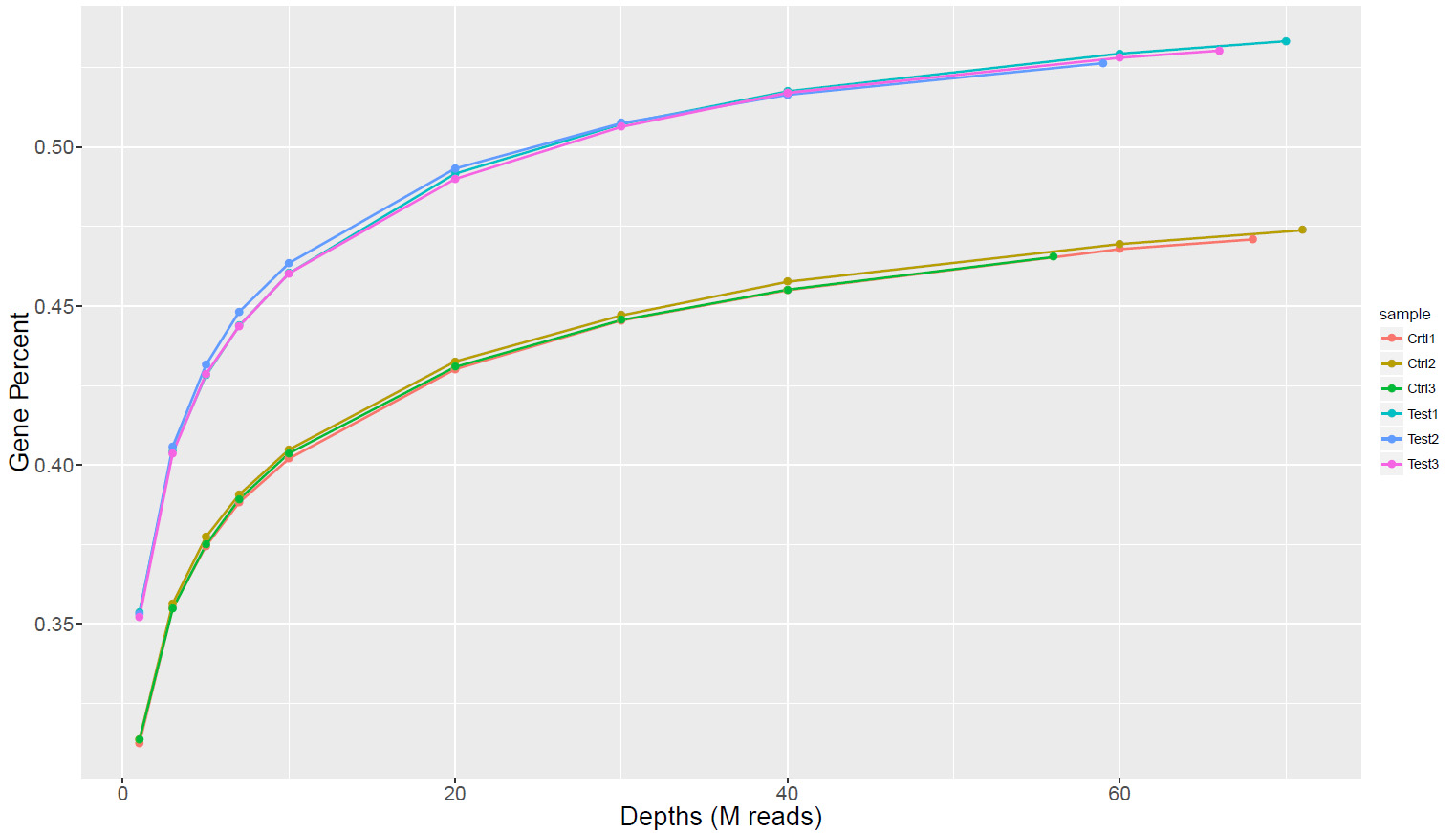

Supplement: Supplementary 1 — Figure S1: saturation analysis of the RNA sequencing data from six libraries. Ctrl1, Ctrl2, and Ctrl3 from SW480 and Test1, Test2, and Test3 from SW480 treated with 2 nM NaB 24 hours. x-axis, sequencing depth; y-axis, proportion of covered genes. [file 1427871.f1.jpg]
